# Familial multiple lipomatosis in a 13-year-old child

**DOI:** 10.11604/pamj.2024.49.129.14033

**Published:** 2024-12-19

**Authors:** Azzeddine Laaraje, Abdelhakim Ourrai

**Affiliations:** 1 Pediatric Department, Faculty of Medicine, The Military Hospital of Instruction Mohamed V, Rabat, Morocco

**Keywords:** Lipomatosis, child, Madelung’s disease, Dercum’s disease

## Image in medicine

A 13-year-old, the only child of her non-consanguineous parents, presented in consultation for multiple subcutaneous masses. The first lesion was detected by the parents at the age of 4 years in the abdomen, after the apparition of other lesions in other parts of the body. The clinical examination revealed the presence of 7 painless, soft, smooth subcutaneous nodules in the abdomen, neck, and arms. The largest was on the right flank of the abdomen, with a maximum diameter of 11 cm. The same symptomatology is found in the father. Ultrasonography and histopathological examination confirmed the diagnosis of lipomatosis. The child´s lipid profile was normal. The diagnosis of familial multiple lipomatosis was retained, in this clinical presentation. It is a rare autosomal dominant disorder; lipomas appear rarely in childhood, symmetrically distributed on the neck, the arms, antebrachial region, the lower limbs, the thoracic, and the abdomen. Face and extremities stay intact. The differential diagnosis included cervical symmetrical lipomatosis (Launois-Bensaude syndrome or Madelung's disease), which manifested a symmetrical lesion mainly affecting the neck and the upper part of the trunk and is associated with chronic alcoholism, and Dercum´s disease, a painful syndrome of the adipose tissue associated with obesity. None of these conditions is probable because the distribution of lipoma was asymmetric and asymptomatic and the child was not obese. Surgical excision is often required to improve cosmetic appearance. In this child, the largest lesions were removed.

**Figure 1:**
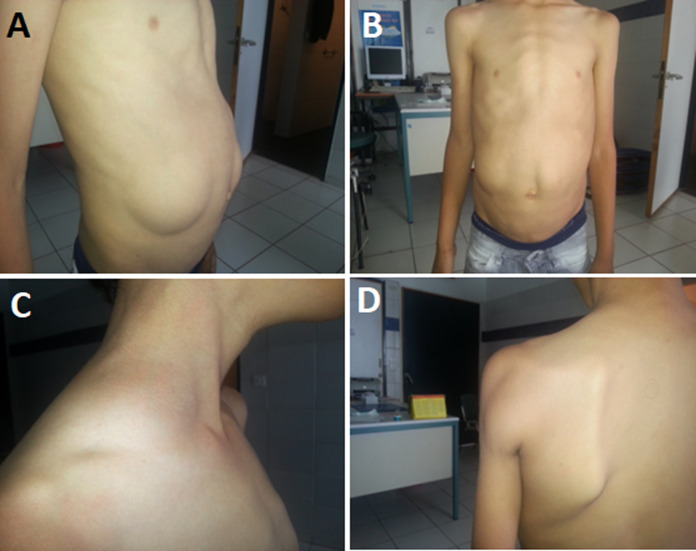
A,B) the abdominal lipomas with a diameter larger than 10 cm; C) lipoma filling the supraclavicular fossa; D) lipoma in the brachial region

